# TCR-triggered extracellular superoxide production is not required for T-cell activation

**DOI:** 10.1186/s12964-014-0050-1

**Published:** 2014-08-01

**Authors:** Aleksey V Belikov, Burkhart Schraven, Luca Simeoni

**Affiliations:** 1 Institute of Molecular and Clinical Immunology, Medical Faculty, Otto-von-Guericke University, Leipziger Str. 44, Magdeburg, 39120, Germany; 2 Department of Immune Control, Helmholtz Centre for Infection Research, Inhoffenstrasse 7, Braunschweig, 38124, Germany

**Keywords:** Reactive oxygen species, ROS, T lymphocyte, T-cell receptor, Proliferation, Cytokine profile, NADPH oxidase, NOX2, gp91phox−/−, Antioxidants

## Abstract

**Background:**

In the last decade, reactive oxygen species (ROS) production has been shown to occur upon T-cell receptor (TCR) stimulation and to affect TCR-mediated signalling. However, the exact reactive species that are produced, how ROS are generated and their requirement for T-cell activation, proliferation or cytokine production remain unclear, especially in the case of primary human T cells. Moreover, several groups have questioned that ROS are produced upon TCR stimulation.

**Results:**

To shed some light onto this issue, we specifically measured superoxide production upon TCR ligation in primary human and mouse T lymphocytes. We showed that superoxide is indeed produced and released into the extracellular space. Antioxidants, such as superoxide dismutase and ascorbate, abolished superoxide production, but surprisingly did not affect activation, proliferation and cytokine secretion in TCR-stimulated primary human T cells. It has been suggested that T cells produce ROS via the NADPH oxidase 2 (NOX2). Therefore, we investigated whether T-cell activation is affected in NOX2-deficient mice (*gp91*^*phox −/−*^). We found that T cells from these mice completely lack inducible superoxide production but display normal upregulation of activation markers and proliferation.

**Conclusions:**

Collectively, our data indicate that primary T cells produce extracellular superoxide upon TCR triggering, potentially via NOX2 at the plasma membrane. However, superoxide is not required for T-cell activation, proliferation and cytokine production.

## Background

T cells play a central role in immune responses and are also involved in the pathogenesis of many diseases. Therefore, a better understanding of the molecular mechanisms regulating T-cell activation is crucial for the development of modern therapeutic tools. In the last decade, it has been shown that TCR triggering leads to the generation of ROS in preactivated T cells, e.g. human and mouse T-cell blasts and Jurkats, resulting in activation-induced cell death (AICD) [1]–[7]. More recently, redox changes upon TCR stimulation were detected in the mitochondria of primary mouse T cells [8]. However, ROS production upon triggering of the TCR in primary human T cells still remains unstudied. Moreover, a number of studies claim that ROS are not produced upon TCR triggering in T cells [9]–[12].

ROS have been proposed to play a role as second messengers [13], by reversibly oxidizing cysteines in phosphatases [14], transcription factors [15], ion channels [16], adaptor molecules [17] and cytoskeleton components [18]. ROS can be inducibly produced by NADPH oxidases [19],[20]. Interestingly, NOX2 [3] and DUOX1 [6] oxidases have been shown to be expressed in T cells.

An indirect evidence that ROS might be important for T-cell functions comes from studies in which various antioxidants were used. Antioxidants were shown to inhibit T-cell proliferation and IL-2 production [11],[21]. However, investigation of the effect of antioxidants on TCR-mediated signalling has given somewhat opposite results. Indeed, antioxidants induced sustained MEK and ERK phosphorylation and promoted mobility shift of Lck upon TCR stimulation in human T-cell blasts and Jurkats [2]. Also, depletion of intracellular glutathione with buthionine sulphoximine led to abrogation of anti-CD3 induced Ca^2+^ flux in Jurkat cells [22] and inhibition of proliferation in primary human T cells [23]. Moreover, it is believed that a reducing milieu, which is promoted by antigen-presenting cells [24], is necessary for T-cell proliferation.

Thus, it is not clear whether ROS is produced and which role oxidation plays in T lymphocytes upon TCR stimulation. In particular, TCR-induced ROS production and its importance in primary human T cells have not been investigated. Therefore, we decided to study ROS production upon CD3×CD28 stimulation in primary human and mouse T lymphocytes. Additionally, we have characterized primary T cells from NOX2-deficient mice to assess whether inducible ROS play any role in T-cell activation. Finally, we investigated the effects of the antioxidant enzymes superoxide dismutase and catalase and the physiological antioxidant molecule ascorbate (Vitamin C) on activation, proliferation and cytokine profile of primary human T cells.

## Results

### TCR stimulation induces the release of extracellular superoxide in primary human T cells

Whether primary human T cells inducibly produce ROS upon TCR ligation has not yet been addressed. Moreover, the exact radical species which are produced and their sources in T cells are not well understood. Therefore, we decided to investigate these issues. We isolated peripheral blood T cells from healthy volunteers. Subsequently, cells were stimulated with CD3 and CD28 mAbs immobilized on microbeads to mimic physiological stimulation [25]. Almost all ROS in biological systems originate from superoxide; therefore, we were interested in the detection of this radical. To this aim, we utilized luminol-based superoxide detection assay Diogenes (from National Diagnostics).

**Figure 1:**
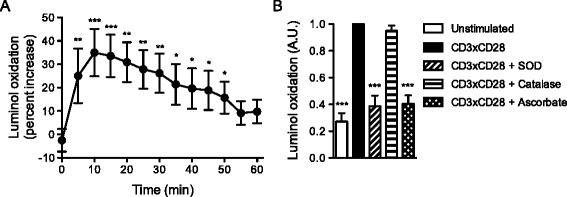
**TCR stimulation induces extracellular superoxide release in primary human T cells. (A)** Primary human T cells were stimulated with CD3xCD28- or isotype-coated microbeads. Superoxide production was measured with Diogenes assay at 5 min intervals. The values indicate the increase in luminescence in CD3xCD28- relative to isotype-stimulated samples. The data show the mean from 16 independent experiments. **(B)** Primary human T cells were stimulated for 10 min with CD3xCD28-coated microbeads alone or in the presence of either SOD, catalase or ascorbate. Superoxide production was measured with Diogenes assay. The values indicate the luminescence signal normalized to stimulated controls. The data show the mean from at least 4 independent experiments.

We indeed detected an increase in luminol oxidation, corresponding to superoxide production, upon CD3×CD28 stimulation as compared to isotype control (Figure 1A). The peak (about 35% increase) in T-cell specific superoxide production occurred 10 min after TCR-triggering and ROS levels decreased gradually afterwards. The superoxide-neutralizing enzyme superoxide dismutase (SOD), but not the hydrogen peroxide-neutralizing enzyme catalase, prevented luminol oxidation, indicating that the detected reactive oxygen specie was indeed superoxide (Figure 1B). These results also suggest that superoxide was released into the extracellular space, as SOD is membrane-impermeable. Interestingly, we were not able to detect extracellular superoxide production upon CD3×CD28 stimulation in human T-cell blasts and Jurkat T cells (data not shown). In summary, primary human T cells produce superoxide upon TCR stimulation and release it to the extracellular space.

### TCR-triggered superoxide production is mediated by NADPH oxidase 2

**Figure 2:**
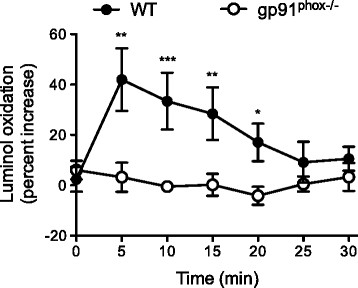
**TCR-triggered superoxide production is mediated by NOX2 in primary T cells.** Splenic T cells from either WT or *gp91*^*phox−/−*^ mice were stimulated with CD3xCD28- or isotype-coated microbeads. Superoxide production was measured with Diogenes assay at 5 min intervals. The values indicate the increase in luminescence in CD3xCD28- relative to isotype-stimulated samples. The data show the mean from 3 independent experiments. 2 WT and 4 *gp91*^*phox−/−*^ mice were used in each experiment.

We showed that T cells produce superoxide upon TCR stimulation. One of the most well-described sources of superoxide is the phagocytic NADPH oxidase 2 (NOX2). Remarkably, NOX2 has been shown to be expressed in T cells [3]. We decided to analyze T cells from NOX2-deficient mice (*gp91*^*phox−/−*^) to investigate whether NOX2 is indeed the source of the superoxide that we detected in our system. Splenic T cells from wild type (WT) mice initiated a similar wave of superoxide production upon CD3×CD28 microbeads stimulation as human T cells, albeit with faster kinetics (Figure 2). Contrary to WT mice, T cells from *gp91*^*phox−/−*^ mice showed no inducible superoxide production upon stimulation (Figure 2). Therefore, these data confirm that NOX2 is indeed activated upon TCR triggering in primary T cells and is responsible for the rapid generation of superoxide.

### Inducible superoxide production is not required for primary human T-cell activation, proliferation and cytokine production

As shown above, both human and mouse primary T cells produce superoxide upon engagement of the T-cell receptor, and this superoxide is released to the extracellular space. In order to investigate the function of superoxide in T cells, we neutralized it by the addition of SOD or the radical-scavenger ascorbate (Figure 1B). Subsequently, we have investigated T-cell activation, proliferation and cytokine production. As superoxide can naturally dismutate to hydrogen peroxide (H_2_O_2_), we have also included samples treated with catalase in our functional assays. SOD, ascorbate and catalase are essential parts of cell-intrinsic antioxidant defense system, and therefore can be safely used without inducing off-target effects.

**Figure 3:**
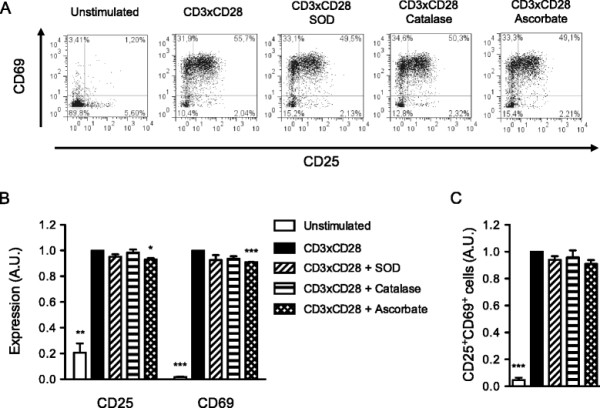
**Extracellular superoxide production is not required for primary human T-cell activation. (A)** Primary human T cells were stimulated with CD3xCD28-coated microbeads alone or in the presence of either SOD, catalase or ascorbate. After 16 hours cells were stained with CD25-FITC and CD69-PE mAbs and analyzed by flow cytometry. The data are representative of 3 independent experiments. **(B)** Quantification of **(A)**. The values indicate the mean fluorescence intensities normalized to stimulated controls. **(C)** Quantification of **(A)**. The values indicate the percentages of CD25^+^CD69^+^ cells normalized to stimulated controls.

Initially, we stimulated primary human T cells with CD3×CD28-coated microbeads for 16 hours in the presence of antioxidants and assessed T-cell activation (Figure 3A). To our surprise, the addition of SOD, ascorbate or catalase had no major effect neither on the expression of CD25 and CD69 activation markers (Figure 3B), nor on the percentage of activated CD25^+^CD69^+^ cells (Figure 3C).

**Figure 4:**
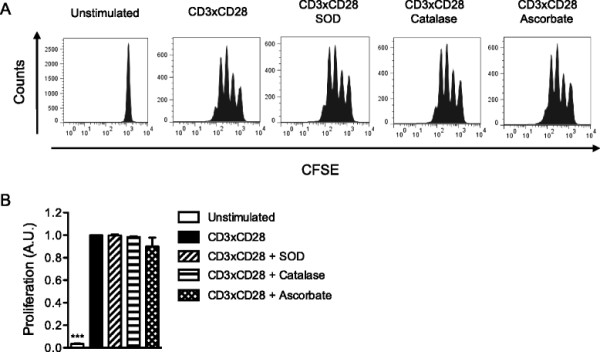
**Extracellular superoxide production is not required for T-cell proliferation. (A)** Primary human T cells were loaded with CFSE and stimulated with CD3xCD28-coated microbeads alone or in the presence of either SOD, catalase or ascorbate. After 72 hours CFSE dilution was analyzed by flow cytometry. The data are representative of 3 independent experiments. **(B)** Quantification of **(A)**. The values indicate the percentages of proliferating cells normalized to stimulated controls.

Next, we investigated proliferation of CD3×CD28 stimulated human T cells in the presence of SOD, ascorbate and catalase using the CFSE dilution assay (Figure 4A). Consistent with the results presented above, we observed normal percentage of proliferating cells 3 days after stimulation in the presence of antioxidants (Figure 4B).

**Figure 5:**
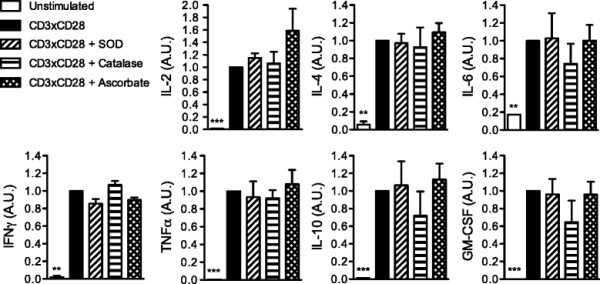
**Extracellular superoxide production is not required for cytokine release by human CD4**^**+**^**T cells.** Human naïve CD4^+^ T cells were stimulated with CD3xCD28-coated microbeads alone or in the presence of either SOD, catalase or ascorbate. After 48 hours, supernatants were collected. Cytokine concentrations were measured using Bio-Plex Pro assay. The values indicate the cytokine concentrations normalized to stimulated controls. The data show the mean from 3 independent experiments.

We then investigated if TCR-triggered superoxide production is involved in the regulation of CD4^+^ T-cell differentiation. Therefore, we stimulated human naïve CD4^+^ T cells with CD3×CD28-coated microbeads in the presence of antioxidants and measured the concentrations of various cytokines in the supernatants after 48 hours using the Bio-Plex system (from Bio-Rad). No significant differences were observed between samples when data were normalized to stimulated controls (Figure 5), as well as when absolute concentrations were used (Additional file 1: Figure S1).

Overall, these data demonstrate that in primary human T cells superoxide production triggered by TCR is dispensable for activation, proliferation and cytokine production.

### NOX2 is not required for T-cell activation and proliferation

**Figure 6:**
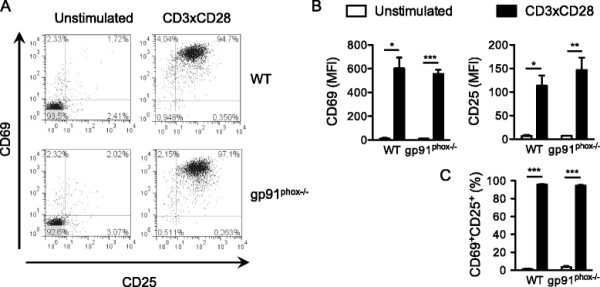
**NOX2 is not required for primary T-cell activation. (A)** Splenic T cells from WT or *gp91*^*phox−/−*^ mice were stimulated with CD3xCD28-coated microbeads. After 16 hours cells were stained with CD25-FITC and CD69-PE mAbs and analyzed by flow cytometry. The data are representative of at least 2 independent experiments. 1 WT and 2 *gp91*^*phox−/−*^ mice were used in each experiment. **(B)** Quantification of **(A)**. The values indicate the mean fluorescence intensities. **(C)** Quantification of **(A)**. The values indicate the percentages of CD69^+^CD25^+^ cells.

**Figure 7:**
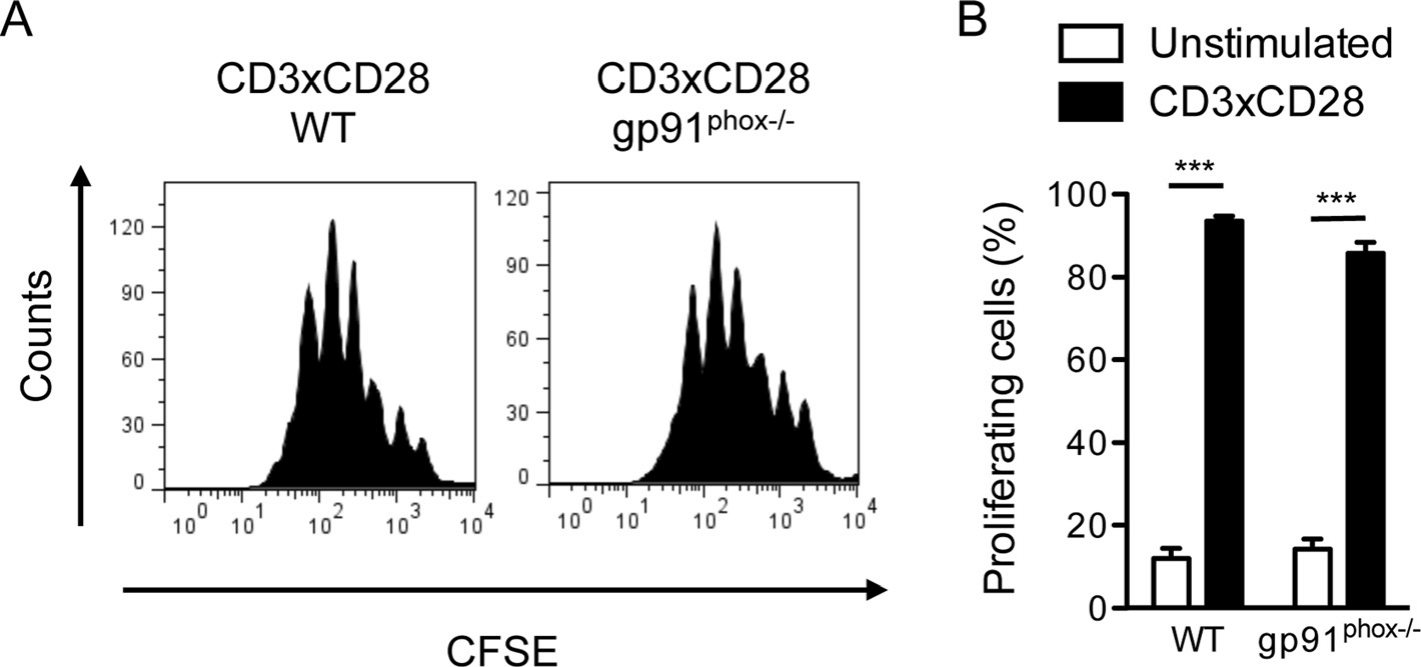
**NOX2 is not required for T-cell proliferation. (A)** Splenic T cells from WT or *gp91*^*phox−/−*^ mice were loaded with CFSE and stimulated with CD3xCD28-coated microbeads. After 72 hours CFSE dilution was analyzed by flow cytometry. The data are representative of 2 independent experiments. 2 WT and 4 *gp91*^*phox−/−*^ mice were used in each experiment. **(B)** Quantification of **(A)**. The values indicate the percentages of proliferating cells.

As shown in Figure 2, *gp91*^*phox−/−*^ mice completely lack TCR-triggered superoxide production. To assess the importance of this reactive oxygen specie in T-cell function, we have investigated the activation and proliferation of *gp91*^*phox−/−*^ T cells upon CD3×CD28 stimulation. In line with our observations on human T cells treated with antioxidants, upregulation of activation markers CD69 and CD25 (Figure 6A, B), percentage of CD69^+^CD25^+^ cells (Figure 6A, C) and proliferation (Figure 7) were not affected in T cells from *gp91*^*phox−/−*^ mice. Altogether, these data show that NOX2 and TCR-triggered superoxide production are dispensable for primary T-cell activation and proliferation.

## Discussion

In this study we showed that primary human and mouse T cells produce extracellular superoxide upon triggering of the T-cell receptor. However, it appears that this reactive oxygen specie is not required for T-cell activation, proliferation and cytokine production. To our knowledge, this is the first study investigating TCR-induced superoxide production and its functional requirement in primary human T cells.

During the last decade, it has been extensively shown that TCR triggering leads to the generation of ROS. However, these studies were performed in preactivated T cells: blasts or Jurkat cell lines [1]–[7]. These cells undergo activation-induced cell death (AICD) upon secondary activation, and the ROS produced are involved in apoptosis regulation and execution. Contrary to these studies, we have not observed any superoxide production in T-cell blasts or Jurkat T cells. This discrepancy may be explained by different methods used to measure ROS production. In our experiments, we used luminol-based assay for detection of extracellular superoxide, whereas in aforementioned studies, DHE (for intracellular superoxide) or DCFDA (for intracellular H_2_O_2_) dyes were used. Moreover, in our study, we employed a system that closely mimics physiological stimulation of T cells by antigen-presenting cells (APCs) [25]. We used anti-CD3 and anti-CD28 mAbs immobilized on microbeads which provide focal stimulation. This cannot be obtained by other systems, including classical stimulation with soluble antibodies, which have been used in other studies. We have shown that, in contrast to soluble stimulation, which results in T-cell unresponsiveness, focal stimulation leads to T-cell activation, proliferation and cytokine production and hence mimics the effects of APCs. Finally, we performed our experiments on primary, non-preactivated cells, which underwent proliferation, not AICD, upon TCR stimulation.

Some reports have suggested that T cells do not produce ROS upon TCR stimulation, and that oxidation of the dyes observed in these studies is an artefact induced by ROS derived from contaminating phagocytic cells [9]–[12]. Those cells can be activated unspecifically by binding of stimulating antibodies to Fc receptors [26]. In our assay, we indeed detected a strong production of superoxide upon addition of microbeads coated with isotype controls, which most likely originated from monocytes contaminating the preparation (Additional file 2: Figure S2). Nevertheless, there was a statistically significant increase in luminol oxidation in T cells stimulated with CD3×CD28 as compared to isotype, indicating an additional T-cell specific superoxide production. Therefore, for ROS measurements in primary T cells purified from blood or tissues, it is crucial to include isotype controls, to discriminate ROS production derived from phagocytic cells.

One of the well-described sources of superoxide is the phagocytic NADPH oxidase. Importantly, the key component of this complex, gp91^phox^, has been shown to be expressed in T cells [3]. However, the same study showed that *gp91*^*phox−/−*^ T-cell blasts were still able to inducibly produce superoxide similarly to WT T cells [3]. In contrary, we observed a complete absence of TCR-triggered superoxide production in primary T cells from *gp91*^*phox−/−*^ mice. The reason for this discrepancy might be the differences in cells, stimulation protocols and superoxide detection methods used. In the aforementioned study, T-cell blasts, stimulated with soluble antibodies, were most likely undergoing AICD, and superoxide from apoptotic mitochondria was detected by intracellular DHE dye. In our study, primary T cells were physiologically stimulated by immobilized antibodies and subsequently have activated NOX2 at the plasma membrane (in case of WT mice) or failed to do so (in case of *gp91*^*phox−/−*^ mice).

In addition to NOX2, other sources of ROS exist in T cells. Mitochondria represent one of those, and have been shown to be involved in AICD of human T-cell blasts [5],[7] and antigen-specific expansion of murine T cells [8]. Additionally, it has been shown that mitochondria translocate to the immunological synapse [27], potentially creating a highly oxidizing environment. Although in our system no superoxide was detected in the absence of NOX2, it is still possible that superoxide is produced in mitochondria upon TCR stimulation, because the Diogenes assay that we used most likely detects only extracellular superoxide. Moreover, mitochondria could produce superoxide at later stages of T-cell activation, whereas we focused on early events following TCR crosslinking. Despite the widespread idea that, upon activation, T cells switch from mitochondrial respiration to aerobic glycolysis [28], it has been shown recently that mitochondria are still the main source of energy and metabolites in activated T cells [8],[29]. Therefore, results obtained from mitochondria-targeted sensors and inhibitors should be interpreted with great caution, as any interference with mitochondrial function is likely to cause severe alterations in cell metabolism or even trigger apoptosis.

To our surprise, T-cell activation and proliferation were not defective in primary T cells from *gp91*^*phox−/−*^ mice. We first thought that the effect could be obscured because of the high activation levels, so we repeated the experiments with low antibody concentration (Additional file 3: Figure S3). However, T cells from *gp91*^*phox−/−*^ mice displayed even slightly higher activation, than WT T cells. This observation could be in line with other studies which, by using mouse models with affected NADPH oxidase function (e.g. *gp91*^*phox*^ and *p47*^*phox*^ knock-out mice), showed an increased severity of T-cell mediated immune diseases, such as arthritis and experimental autoimmune encephalomyelitis [30]–[32]. However, the exact mechanisms responsible for this effect are not clear. Moreover, these mice have a defect in ROS production by phagocytic cells as well, which could have contributed to the development of these autoimmune diseases. In addition, it has been shown that CD4^+^ T-cell blasts from *gp91*^*phox−/−*^ mice display skewing of cytokine profile towards Th1 [3].

In agreement with our results from *gp91*^*phox−/−*^ mice, natural antioxidants did not affect primary human T-cell activation and proliferation. This appears to be in contrast with some previously published data showing that antioxidants inhibit T-cell proliferation and IL-2 production [21]. However, non-naturally occurring antioxidant compounds were used in that study. Those are likely to induce various off-target effects. Interestingly, one study showed that concentrations of ascorbate required to inhibit proliferation or IL-2 production in T cells are two orders of magnitude higher than those required to inhibit ROS production [11].

Despite the fact that superoxide is produced upon TCR stimulation, it appears to be dispensable for T-cell activation, proliferation and cytokine production. This raises the question: why do T cells need to produce superoxide? An intriguing possibility is that extracellularly released superoxide serves as a feedback messenger to antigen-presenting cell, to signal successful activation. Indeed, a tightly regulated crosstalk between dendritic cells and T cells has been demonstrated [24],[33]. Further studies investigating T cell-APC interactions *in vitro* or *in vivo* are required to assess this hypothesis.

## Conclusions

In summary, we showed that triggering of the T-cell receptor on primary human and mouse T cells induces rapid extracellular superoxide production, potentially via NADPH oxidase 2. Nevertheless, the functional importance of this event remains elusive, as we have not found any significant defects in the expression of activation markers, proliferation and cytokine secretion when superoxide production was abolished.

## Methods

### Ethics

Approval for these studies was obtained from the Ethics Committee of the Medical Faculty at the Otto-von-Guericke University, Magdeburg, Germany, with the permission number [107/09]. Informed consent was obtained in writing in accordance with the Declaration of Helsinki. All experiments involving mice were performed according to the guidelines of the State of Sachsen-Anhalt, Germany.

### Human T-cell purification and culture

Peripheral blood mononuclear cells were isolated by Ficoll gradient (Biochrom AG) centrifugation of heparinized blood collected from healthy volunteers. T cells were further purified by non-T cell depletion using human pan T-cell isolation kit and AutoMACS (all from Miltenyi Biotec). Purity of T cells, determined by flow cytometry, was routinely more than 96%. T cells were cultured at 10^6^ cells/ml, 37°C and 5% CO_2_ in RPMI 1640 medium supplemented with stable glutamine (Biochrom AG), 10% fetal calf serum (PAN Biotech) and 2 μg/ml ciprobay (Bayer).

### Mouse T-cell purification and culture

*gp91*^*phox−/−*^ mice were a kind gift from Dr. Katrin Breitbach (Friedrich Loeffler Institute of Medical Microbiology, Ernst-Moritz-Arndt University, Greifswald). Wild type control C57BL/6J^Bom^ mice were obtained from Taconics. Mice were kept in pathogen-free conditions (SPF). Spleens from mice were passed through a fine mesh filter (BD Falcon) to obtain a single-cell suspension. T cells were purified by non-T cell depletion using mouse pan T-cell isolation kit and AutoMACS (all from Miltenyi Biotec). Purity of T cells, determined by flow cytometry, was routinely more than 96%. The cells were cultured at 10^6^ cells/ml, 37°C and 5% CO_2_ in RPMI 1640 medium supplemented with stable glutamine (Biochrom AG), 10% fetal calf serum PAN Biotech), 2 μg/ml ciprobay (Bayer) and 50 μM β-mercaptoethanol (Sigma Aldrich).

### T-cell stimulation

T cells were stimulated with immobilized CD3×CD28 mAbs as previously described [25]. Briefly, SuperAvidin™-coated polystyrene microspheres (Bangs laboratories, Inc., Ø ~ 10 μm, binding capacity: 0,02-0,04 μg biotin/mg) were coated with biotinylated CD3 (clone UCHT1) and CD28 (clone CD28.2) mAbs (10 μg/ml each, BioLegend) for 30 min at 37°C in PBS Dulbecco (Biochrom AG). Antibody-coated microspheres were washed three times, resuspended in PBS at 10^8^ beads/ml and incubated with T cells in a 1 bead per cell ratio. Microspheres coated with 20 μg/ml of biotinylated mouse IgG1κ (eBioscience) were used as a control.

Stimulations in the presence of 100 U/ml superoxide dismutase from bovine erythrocytes, 1000 U/ml catalase from *Corynebacterium glutamicum* and 100 μM L-ascorbic acid (all from Sigma-Aldrich) were performed by pre-incubating T cells for 30 min with the compounds before stimulation.

Mouse T cells were stimulated with CD3 (clone 145-2C11) and CD28 (clone 37.51) mAbs (10 μg/ml each, both from BD Pharmingen) immobilized on microspheres as described above. Microspheres coated with biotinylated hamster IgG1κ and IgG2λ1 (10 μg/ml each, both from BD Pharmingen) were used as a control.

### Superoxide production assay

For superoxide detection the Diogenes Cellular Luminescence Enhancement System (National Diagnostics) was used according to the manufacturer’s instructions. Briefly, T cells were centrifuged, resuspended at the density of 10^7^ cells/ml in a prewarmed serum-free phenol red-free RPMI 1640 medium (Gibco) and aliquoted to black polystyrene flat bottom 96-well plate (Costar, Corning Inc., 100 μl suspension per well). Diogenes Reagent and Diogenes Activator were freshly mixed in a 1:9 ratio and aliquoted to the same plate (50 μl per well). Cells were then stimulated as described above and luminescence was repeatedly measured with 5 min intervals for the total of 60 min using TriStar LB941 multimode reader (Berthold technologies), keeping cells at 37°C.

### Activation assay

T cells were seeded onto flat bottom polystyrene 48-well plates (Costar, Corning Inc.) at 10^6^ cells/ml in a volume of 500 μl per well and stimulated as described above. After 16 h, cells were stained with FITC- or PE-labeled mAbs against CD25 and CD69 (BD Pharmingen, BioLegend) and analyzed by flow cytometry using a FACSCalibur and CellQuest software or BD LSRFortessa and FACSDiva Software 6.1.3 (all from BD Biosciences), and FlowJo 7.5.5 (Tree Star, Inc.).

### Proliferation assay

T cells were centrifuged, resuspended in 1 ml of PBS Dulbecco (Biochrom AG) and labeled with 2,5 μM CFSE (Invitrogen) for 15 min at 37°C. Then cells were washed twice, resuspended in medium at the density of 10^6^ cells/ml, seeded onto flat bottom polystyrene 48-well plates (Costar, Corning Inc.) in a volume of 500 μl/well and stimulated as described above. Cells were then cultured for 72 h and proliferation was assessed by CFSE dilution using FACSCalibur and CellQuest software or BD LSRFortessa and FACSDiva Software 6.1.3 (all from BD Biosciences), and FlowJo 7.5.5 (Tree Star, Inc.).

### Cytokine assay

Human naïve CD4^+^ T cells were purified from peripheral blood mononuclear cells by non-T cell depletion using human naïve CD4^+^ T-cell isolation kit and AutoMACS (all from Miltenyi Biotec). T cells were resuspended in serum-free X-VIVO 15 medium supplemented with gentamycin (Lonza), seeded onto flat bottom polystyrene 48-well plates (Costar, Corning Inc.) at 10^6^ cells/ml in a volume of 1 ml per well and stimulated as described above. After 48 h supernatants were harvested, supplemented with 0,5% bovine serum albumin (Sigma-Aldrich) and frozen. Later, cytokine concentrations in the supernatants were measured using Bio-Plex Pro assay on the Bio-Plex 200 system (all from Bio-Rad) according to the manufacturer’s protocol.

### Statistics

Statistical analysis was performed using GraphPad Prism 5 (GraphPad Software, Inc.). *P* values were determined by two-tailed Student’s *t* test. *P < 0.05, **P < 0.01, ***P < 0.001.

## Abbreviations

AICD: Activation-induced cell death

APC: Antigen-presenting cell

CFSE: Carboxyfluorescein succinimidyl ester

DCFDA: 2′,7′-dichlorofluorescin diacetate

DHE: dihydroethydium

FITC: Fluorescein isothiocyanate

NOX2: NADPH oxidase 2

PE: Phycoerythrin

ROS: Reactive oxygen species

SOD: Superoxide dismutase

TCR: T-cell receptor

WT: Wild type

## Competing interests

The authors declare that they have no competing interests.

## Authors’ contributions

AVB participated in the design of the study, carried out all the experiments, performed the statistical analysis and drafted the manuscript. BS participated in the design of the study and helped to draft the manuscript. LS conceived of and coordinated the study and helped to draft the manuscript. All authors read and approved the final manuscript.

## Additional files

## Supplementary Material

Additional file 1: Figure S1. Extracellular superoxide production is not required for cytokine release by human CD4^+^ T cells. Human naïve CD4^+^ T cells were stimulated with CD3xCD28-coated microbeads alone or in the presence of either SOD, catalase or ascorbate. After 48 hours, supernatants were collected. Cytokine concentrations were measured using Bio-Plex Pro assay. The values indicate the absolute cytokine concentrations.The data show the mean from 3 independent experiments. Click here for file

Additional file 2: Figure S2. TCR stimulation induces extracellular superoxide release in primary human and mouse T cells. (**A** and **B**) T cells were stimulated with CD3xCD28- or isotype-coated microbeads. Superoxide production was measured with Diogenes assay at 5 min intervals. The values indicate the increase in luminescence in CD3xCD28- or isotype-stimulated samples relative to unstimulated samples. **(A)** Primary human T cells were used. The data show the mean from 16 independent experiments. **(B)** Splenic T cells from either WT or *gp91*^*phox−/−*^ mice were used. The data show the mean from 3 independent experiments. 2 WT and 4 *gp91*^*phox−/−*^ mice were used in each experiment. Click here for file

Additional file 3: Figure S3. NOX2 is not required for primary T-cell activation. Splenic T cells from WT or *gp91*^*phox−/−*^ mice were stimulated with CD3 antibody, immobilized on culture plates in concentrations 5 μg/ml or 0,15 μg/ml. After 16 hours, cells were stained with CD25-FITC and CD69-PE mAbs and analyzed by flow cytometry. The data are representative of 2 independent experiments. The values indicate the mean fluorescence intensities or the percentages of CD69^+^CD25^+^ cells. Click here for file
