# Supplementary figures and images for: TCR-triggered extracellular superoxide production is not required for T-cell activation
Source: Cell Commun Signal. 2014 Aug 1;12:50. doi: 10.1186/s12964-014-0050-1 (PMC4237797; doi:10.1186/s12964-014-0050-1)

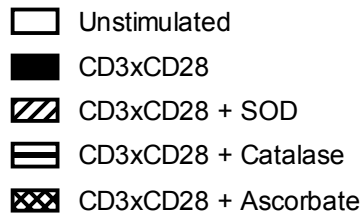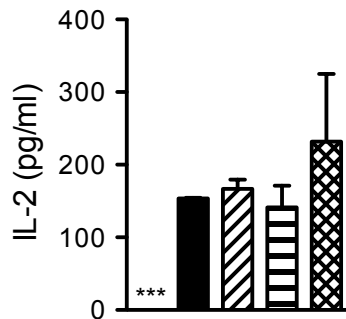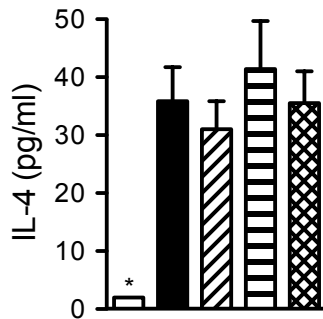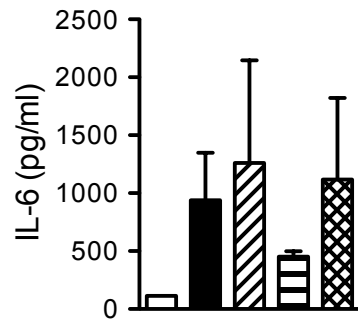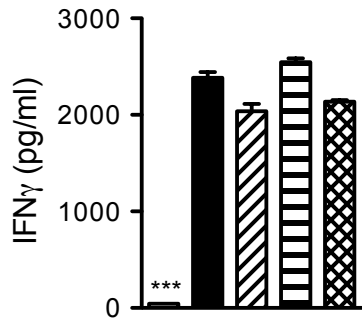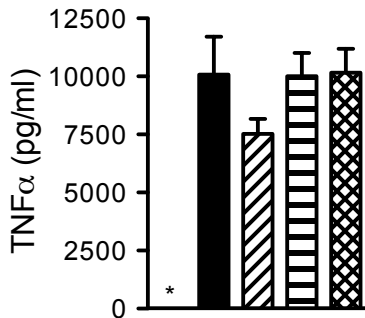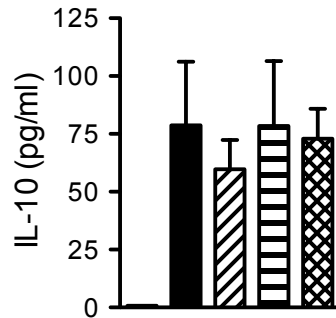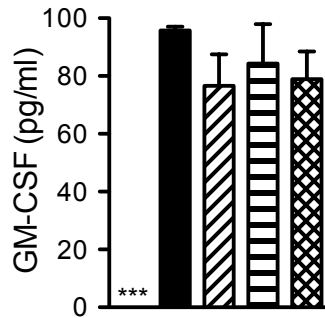

Supplement: Additional file 1: Figure S1. — Extracellular superoxide production is not required for cytokine release by human CD4+ T cells. Human naïve CD4+ T cells were stimulated with CD3xCD28-coated microbeads alone or in the presence of either SOD, catalase or ascorbate. After 48 hours, supernatants were collected. Cytokine concentrations were measured using Bio-Plex Pro assay. The values indicate the absolute cytokine concentrations.The data show the mean from 3 independent experiments. [file s12964-014-0050-1-S1.pdf]

A

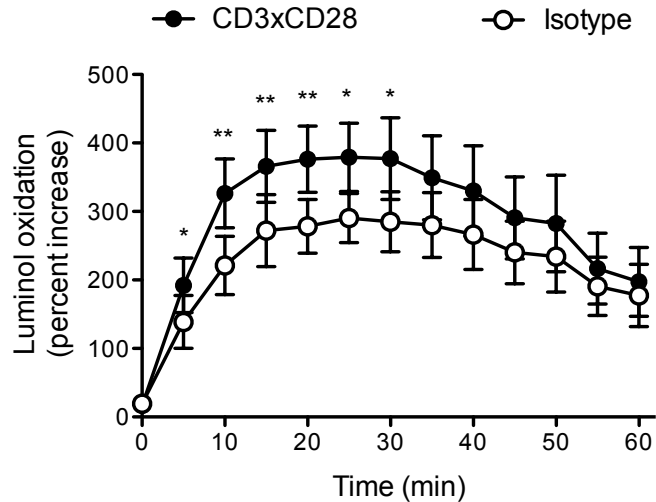

B

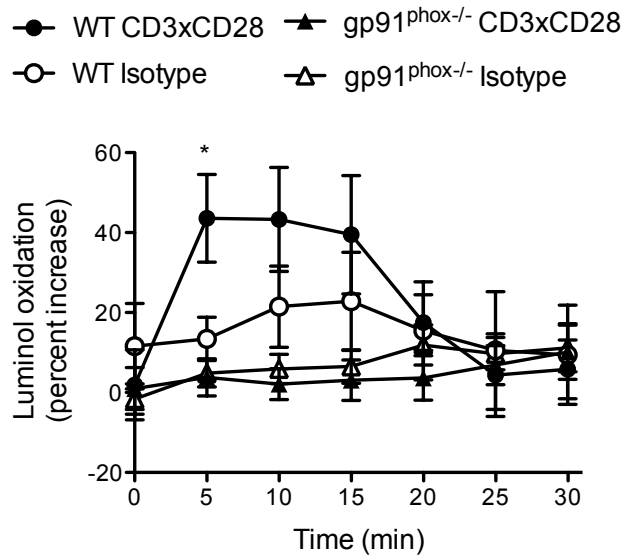

Supplement: Additional file 2: Figure S2. — TCR stimulation induces extracellular superoxide release in primary human and mouse T cells. (A and B) T cells were stimulated with CD3xCD28- or isotype-coated microbeads. Superoxide production was measured with Diogenes assay at 5 min intervals. The values indicate the increase in luminescence in CD3xCD28- or isotype-stimulated samples relative to unstimulated samples. (A) Primary human T cells were used. The data show the mean from 16 independent experiments. (B) Splenic T cells from either WT or gp91phox−/− mice were used. The data show the mean from 3 independent experiments. 2 WT and 4 gp91phox−/− mice were used in each experiment. [file s12964-014-0050-1-S2.pdf]

□ Unstimulated

■ 5  $\mu\text{g/ml}$  CD3

▨ 0,15  $\mu\text{g/ml}$  CD3

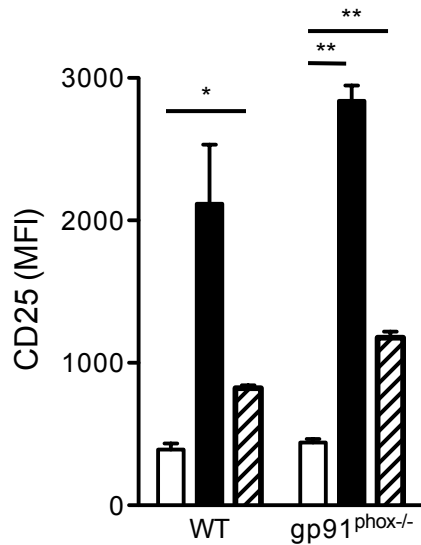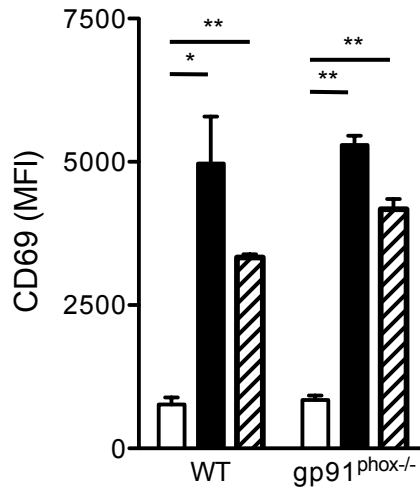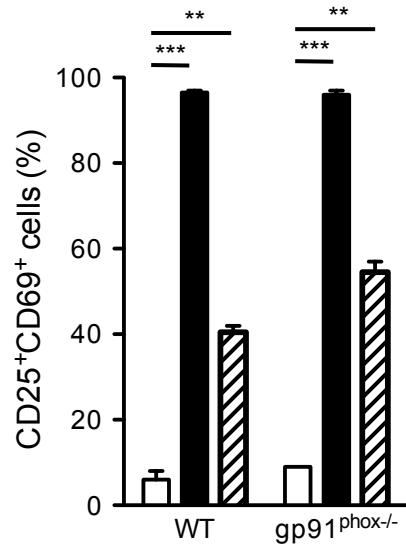

Supplement: Additional file 3: Figure S3. — NOX2 is not required for primary T-cell activation. Splenic T cells from WT or gp91phox−/− mice were stimulated with CD3 antibody, immobilized on culture plates in concentrations 5 μg/ml or 0,15 μg/ml. After 16 hours, cells were stained with CD25-FITC and CD69-PE mAbs and analyzed by flow cytometry. The data are representative of 2 independent experiments. The values indicate the mean fluorescence intensities or the percentages of CD69+CD25+ cells. [file s12964-014-0050-1-S3.pdf]
